# On the Unity of the Human Species

**Published:** 1860-10-01

**Authors:** A. Brierre de Boismont, H. J. Manning


### Art. II.—ON THE UNITY OF THE HUMAN SPECIES,

### CONSIDERED WITH RELATION TO THE AMELIORATION OF RACES BY EDUCATION AND INTERMARRIAGE.*

By A. BRIERRE DE BOISMONT.

(*Translated from the French by H. J. Manning, Esq.*)

When I took up my pen to write the history of hallucinations, my wish was to enter a protest against the scientific opinion which makes Socrates, Pascal, Joan of Arc, and many others, downright madmen. The motive which sends me into the lists now is the instinctive repugnance which I feel to the doctrine of the inequality of races. I find it impossible, indeed, to look upon all the inhabitants of the different countries of the earth otherwise than as members of the same family. Isolation, misery, famine, conquest, emigration, ignorance, &c., have modified original types, made them stationary, retarded or even degenerated them; but the attentive observer perceives that it only wants devotedness and will to remedy this state of things. Examples abound to prove that colonies, apparently the least favoured, may be raised from their apparent decay, either by the aid of social relations, or by means of intermixture. Such will be the object of this work.

Writers have supposed that they have found a decisive argument, from having seen in the bazaars of Cairo or Damascus, the colossal Circassian; the Egyptian, of shorter stature and arched nose; the Nubian, of the colour of violet, but with an agreeable face, the nose short and small, the teeth fine and even; the Turk, with white and transparent skin; the Negro, with crisp hair, flat nose, high cheek-bones; the Fellah, olive-coloured; the Bedouin, almost as black as the Nubian, but of tall stature, aquiline nose, and royal mien. Recalling this quotation from an illustrious naturalist, the author of the *Plurality of Races*, who promises to bear worthily his father's name, adds: "And yet all these men, so opposite to one another, live and have lived for centuries at the distance of a few leagues, and almost under the same sky!"

We might remark that the geographical distance of most of these people from one another is not so inconsiderable as M. Pouchet thinks. We might also add that he has grouped in this picture only the extremes of each type; but we prefer answering

* Read before the Société Médicale du Pantheon, 14 March, 1860, *apropos* of a discussion upon the different races of mankind, occasioned by "the remarkable work" of M. Hipp. Lamarche, *La Politique et les Religions, Etudes d'un Journaliste* (Paris, 1859), one of the chief arguments of which is summed up by the author in these terms: "I believe with our great masters in the unity of the human species, in the superiority of the Caucasian race, shown by continual progress, and in the possible amelioration of races less thoroughly distributed by intermixture with our own."

him by a similar group of the different peoples of the European race. Examine the German, the Russian, the Spaniard, the Italian, the Frenchman, the Englishman, and you will be struck by the difference in feature, language, and habits presented by each of these races. Nay more: the same country will offer the most striking dissimilitude between its different divisions. To quote but one example of this—the Piedmontese could not be mistaken for a Neapolitan. Yet who would dispute the common origin of all these nations?

These preliminaries set forth, we have only now to lay down our arguments.

An examination of the physical, physiological, and psychological characteristics of our species—such is the plan to which I have limited myself; yet I do not hesitate to confess that, as I advanced in the study of the question, my own inefficiency alarmed me, and that, but for the engagement I had entered into with the Society to which for some time I have had the honour of belonging, I should have abandoned the task. When I saw among the adversaries of the unity of the human species such men as Linnæus, Geoffroy St. Hilaire, Richard Owen, and other names full of energy, youth, and science, I felt that I had nothing new to show, and that my inquiries could only be analytical and critical, which placed me in a marked degree of inferiority; but led on by my philosophical convictions, fortified by certain anthropological observations, calling to my help Blumenbach, Cuvier, Humboldt, Flourens, and Professor Godron (of Nancy), who has written a book* rich in facts, and from which I have borrowed extensively, I followed up my undertaking; and it is the result of my labour which I present to the Society, and for which I claim every indulgence.

A circumstance which I had frequently observed, and which all medical men may observe, had also predisposed me in favour of the unity of the human race. Nothing is more common than to find in the same family handsome, well-made children, and others ugly and ill-formed. The configuration of the cranium offers sometimes the most opposite shapes. The colour of the skin is often variable. By the side of the fairest skins are seen others of yellow or brown tint, approaching almost to the foreign type. The difference in intellect is not less decided: the highest qualifications spring up in all their brilliancy side by side with the most confirmed idiocy. Yet no one outside the circle in which they are produced takes it into his head to seek for an explanation of dissimilitudes in appearance so alarming. Why should the human species escape this great family law?

* *De l'Espèce et des Races dans les Etres Organisés, et spécialement de l'Unité du Genre Humain.* Two vols. Paris, 1859.

Doubtless the four principal races among whom the world is divided—the white, the black, the tawny, and the red—present numerous differences; but there is one primitive law which must not be lost sight of, and that is the reproduction of all these races, one by another. This physiological fact, laid down by Buffon, and demonstrated by M. Flourens, is now incontrovertible, and constitutes the species. Whatever analogy is presented by certain species, however possible their intermixture may be, sterility is sooner or later the result. Other considerations of the highest importance may be summoned in favour of the unity of the human race.

A profound study of secondary meteorological influences, of the important influence exercised by the mode of life, of profound modifying causes, of inheritance, of amalgamation, of all the circumstances which go to form customs, habits, religious laws, and the moral aspect of a people, explains the repeated varieties to be observed in the physical characteristics and intellectual development of a people, and leads to the recognition of but one family in these men of such different colour and appearance.

This doctrine of the unity of the human race, one of the most beautiful ornaments of Christianity and philosophy (the proof of which is, that it has caused barbarity and cruelty, formerly permanent, to be acts instantaneously and momentarily branded): this doctrine penetrates the conscience little by little; and, in spite of the opposition it encounters, we foretel the time when it will become the code of humanity.

We are well aware that it seems at first sight difficult, not to say impossible, to place on the same level, and to consider as belonging to the same family, the fair-skinned Caucasian type, with its oval face, broad forehead, Greek profile, aquiline nose, and straight front teeth, and the Negro type, with its black skin, frizzled hair, receding head, narrowed at the top, and long jawbone. But this parallel is a style of argument which is merely begging the question. We have taken the two extremes of beauty and ugliness, and left in the background the intermediate series which unites the two ends of the chain.

Let us take the most striking difference, viz., the colour. Certainly nothing could be more opposite than the ebony black of the Negro of the Guinea coast, and the rosy white of the European. But, according to Dr. Charles Livingstone,* one of the most able explorers of Africa, this hideous colour, commonly placed as a sign outside a tobacco shop, only exists among the lowest class of the population. During his extensive wanderings across Central Africa he has noticed the black tinged with olive,

* Livingstone: *Explorations dans l'Afrique Australe, ouvrage traduit de l'Anglais.* Par Mme. Loreau. Paris, 1859.

the olive tint less deep, the bronze tint, and the coffee colour; the black colour is specially marked in the districts which are hot and damp. Prichard has remarked that there are tribes in Africa with a brown skin, with chocolate colour, or simply sunburnt—(3rd edit. vol. ii. p. 158). Schreber declares that there are in Africa and Madagascar yellow Negroes and red Negroes with the same kind of hair.* And, lastly, Prichard points out the "Gallas Edjows" as almost white, though living under the equator.

If we confine ourselves to this fact, this fair colouring of the Negro may appear astonishing, but it is not peculiar to this race. The Touaregs, pirates of the Sahara, are white in certain countries, according to General Daumas, and it is not even very rare to meet among them fair-complexioned women with blue eyes.† In other districts, according to Heeren, they have the tawny and even black skin, without the crisp hair or the features of the Negro. These Touaregs only marry among themselves. Lastly, Abel de Rémusat reports that here are in the provinces of Central China women of fair complexion with the same varieties of tint that are seen among the women of Central Europe.‡

More recent documents, published by Baron H. Aucapitaine, and inserted in the *Revue et Magasin de Zoologie,* give the following information respecting the colouring of the skin among the Negroes of Kabylia:—

"M. d'Abaddie, known by his travels in Abyssinia, has just addressed to M. Quatrefages§ a letter relating to a very curious anthropological fact, viz., the effect of an exclusively animal diet upon the colour of Negroes. The learned French traveller tells us that in the south of Nubia those blacks who live wholly upon meat have a clearer tint than the other tribes whose diet is exclusively vegetable. Reading this remark led me to a similar observation with regard to the Negroes of Kabylia. Meat in Kabylia is very dear; it is a luxury which the Berber does not allow himself every day. But the Negroes, who are all butchers, feed constantly on the remains of animals which they sell in the markets. Their life, like those of whom M. d'Abaddie speaks, is passed amidst blood and fleshy vapours; they have a very clear tint, though preserving, both men and women, the frizzle hair and all the characteristics of the race of Haoussa."

["Till now, I had always attributed this fact to the mixed blood of the Kabyles and the cold climate. I happened to be at Tamda-el-Blat, among the Beni-Djennad, when I received the *Bulletin de la Société de Géographie;* I was able at once to get information among the numerous freed men who reside in this village, and I found that the Negroes only marry *among each*

* *Historia Naturalis Quadrupedum,* vol. i. pp. 14, 15.

† *Voyage au Grand Désert du Sahara.*

‡ *Recherches sur les Langues Tartares,* 1820.

§ *Bulletin de la Société de Géographie,* 1859, vol. xiv., p. 179.

*other*, although they are looked on in Kabyle society, which is essentially democratic, as fellow-citizens equal to the rest.

Must we attribute this fact to degenerate blood, the result of repeated marriages between members of the same race? I think not. It must be owing then, as M. d'Abaddie says, to their feeding continually on meat, and to the contact with bleeding flesh which they are constantly dragging and moving about. This appears to me to be a very interesting question in an anthropological point of view, and one which deserves close investigation."]*

The Negro race then may show skins of very different colour; and this fact is observable also in other races.

The Abyssinians, who preserve evident characters of their semitic origin, are both black, brown, and almost white.

The Jews themselves have not preserved everywhere their primitive colour. In the northern countries of Europe they are white; in Germany many of them have red beards; in Portugal they are tawny. In the province of Cochin China, where a number of them have settled, they have black skins, though they do not contract marriages with foreigners. Prichard† says, that there is also at Mattachéri a colony of white Jews, who are called in India Jerusalem Jews. And, lastly, there are black Jews dwelling in Africa, in the kingdom of Haoussa.

Thus great varieties of colour have been produced among this people during eighteen centuries, but no change has occurred in their cast of feature, habits, or ideas. Under a black skin or a white, observes General Daumas, in Soudan, in the Sahara, or the sea-coast towns, everywhere Jews have the same instincts, and the twofold aptitude for languages and commerce.‡

Colour, then, is not a fixed characteristic. It may vary among members of one and the same race, or of one and the same tribe. And this is frequently observable also in domestic animals.

We are all aware that colouring of the skin is due to pigmentary secretion, that it is present in all races, and that though very limited among Europeans, it is plainly seen on the nipple. M. Flourens showed it us very well developed in a French soldier who had lived a long time in Algiers. He has discovered it among whites by means of the microscope. And he has proved that in the fœtus of the Negro, as in that of the white, there is no trace of pigment. In a communication lately made to the Anthropological Society (3rd Nov. 1859), Dr. Gubler reports, that, wishing to compare the brain of a Negro in the service of

* *Moniteur Universel* du 22 Mars, 1860, note de M. le Baron Aucapitaine, insérée dans la *Revue et Magasin de Zoologie.*

† *Histoire Naturelle de l'Homme.* Paris, 1843.

‡ *Le Grand Désert*, p. 244.

M. Rayer, who died at La Charité, with that of white men, specially with regard to the internal dark colouring so marked in the black race, he placed at intervals on the same table brains procured from fair and dark complexioned subjects. He was then enabled to determine that the substance was paler in the first than in the second, and that the deepest colouring in the latter was of the nature of pigmentary secretion. And in these latter subjects it is not only in the cerebral substance that the colouring matter is deposited. Analogous deposits are sometimes met with in the pia mater surrounding the protuberance. M. Virchow, according to M. Brown-Séquard, has often seen pigmentary colouring under the pia mater of white men, and notably under the medulla oblongata; and it is probable that this matter, kept in reserve, so to speak, may fill some part in the economy. It follows from these facts that colouring matter is not so rare in the European as has been supposed.

Numerous causes have naturally been sought, to account for the production of colour. Climate and heat have been most frequently cited. And truly if we ascend from Norway to the Equator, we see the skin changing gradually; from white becoming sunburnt, then brown, and lastly black, in Soudan. But if the climate is the cause of these variations, the same causes ought to produce everywhere the same effects. But yet in Europe the Laplanders, with their tawny complexion, form an exception; if this depends on cold, why have the Icelanders so white a skin, with blue eyes and light hair?

Besides these, there is an army of facts which prove that the colour of the skin has nothing to do with the heat of the climate. Thus in Asia may be seen brown-skinned Calmucks side by side with Georgians and Circassians, who are so remarkable for the whiteness of their skin. Not far from the inhabitants of Cashmere, who are white or nearly so, and under the same latitude, we find the Nepaulese, who, notwithstanding the great elevation of their mountains and their temperate climate, have a black skin; whereas the neighbouring Bengalese, who live in the plains and more to the south, have a skin only coffee-coloured.

The Portuguese, who have been settlers on the Guinea coast since the fifteenth century, and on the Mozambique coast since the sixteenth, have not lost their original colour.*

The Arabs, who inhabited these same coasts many centuries before the arrival of the Portuguese, have not taken the Negro colour.

We should have the same observations to make with regard to direct heat, the hygrometric state of the air, &c., for with these

* Salt: *Voyage en Abyssinie, trad.*, vol. i., pp. 72—94.

two conditions we should find facts similar to the foregoing; so that it is with us an established principle that in analyzing the chief elements of the influence of climate, we arrive at the conclusion that this influence is always secondary.

To what then shall we attribute these varieties in colour presented by the numerous races of man? In all probability to the same internal causes, still unknown, which produce them in domestic animals, and among which "albinism," "erythrism," and "melanism," play an important part. These three different colours, which are modifications of the pigmentary secretion, are to be observed in a number of animals which enjoy perfect health, and are able to reproduce their species. To quote only one instance, there are in India, white, red, and black elephants. Consequently we believe that M. Isidore Geoffroy St. Hilaire was right in supposing that the absence of pigment, or, according to us, its extreme scarcity (which constitutes albinism), was the normal state of animals naturally white.* With regard to erythrism and melanism, their existence with health is still less doubtful. The first of these colours, which is also observable in animals, is the normal state of the "Red Skins" of America. It was the colour of the ancient Egyptians. We had in our establishment the wife of a superior officer, of Coptic origin, who recalled in a striking manner the figures on the ancient monuments. Melanism, so common with domestic animals, forming among them permanent races, gives to the skin characters which do not differ from those observed in the cutaneous apparatus of the black man; so that we may look upon this latter as afflicted with normal melanism.

Melanism, like albinism and erythrism, may be partial and congenital. Thus, with certain white women, the areola of the nipple is entirely black. Blumenbach has described a portion of the skin of the abdomen of a beggar which was as black as the skin of an African. Camper tells of a woman who, whenever she became pregnant, showed a development of pigment which invaded the whole abdomen; and a similar instance is known, where the melanism extended from the neck to the lower part of the body.

We have then in the internal modifications which the secretion of colouring matter undergoes, whether it disappears or varies in its elements, or whether it sustains an increase more or less considerable, an explanation of the different colourings of the skin. As to the time when they took place, we believe with M. Godron, that it extends back to the origin of different nations: the important thing for us to know is that they may be produced in our

* *Histoire Générale et Particulière des Anomalies de l'Organisation chez l'Homme et les Animaux*, vol. i., p. 317. Paris, 1832.

race, and that these varieties of colour are not sufficient to constitute a difference of race.

We now come to four other characteristics set down as special; the frizzled and woolly state of the hair, the obliquity of the eyes, the shape of the skull, and the features of the face; because, taken separately, there is something decided in them, while they cease to be exceptional when studied in the series of beings.

One important remark which we have to make with regard to the hair of Negroes, is that, as Prichard has shown, it is not different from that of other men, and that it bears no resemblance whatever to wool. Its frizzly disposition has more than one exception. Thus the Danish missionary Isert met on the Gold Coast a small tribe of Negroes, whose hair was a foot and a-half long.* Barbot relates that the Fentis, the Ashantis, the Aguapins, and the Intas, have frequently curly hair long enough to reach to the shoulders. And lastly, Prichard adds, that the hair of different Negro tribes presents every possible gradation, from the woolly head of hair, to the crisp, and even the wavy.†

The first sight of a Chinaman, with his slanting eyes, gives one a strange impression, and we should be tempted to believe in the idea of a different species of man. But this striking characteristic is not general in China. Thus at Canton, and in the towns in the north of China, this characteristic is even exceptional, especially among the men. We were present one evening at a performance in the Cirque de l'Impératrice, when fourteen foreigners came and sat near us. It was the Siamese Embassy. We examined closely their faces during the whole performance, and found that several of them had not slanting eyes. When Abel Rémusat received at the Bibliothèque the young Chinamen who were to preach the Christian religion, one of them struck us by the regularity of his features and the shape of his face, which approached closely the European type. Nor is the obliquity of the eyes peculiar to the Chinese, Japanese, or Mongols: it is found among the Caribs of South America‡ and the Botocudos of Brazil.§ The resemblance is striking when we meet at Rio a Chinaman and a Botocudo. Livingstone has made known this disposition of the eyelids among the tribes of Southern Africa.—(p. 493.) This obliquity of the eyes is in reality an obliquity only of the eyelids, the external angle of which is more raised. Lastly, we have several times noticed it in a striking degree among Europeans.

The difference in shape of the skull has been thought a strong argument. It is clear that among savage tribes it is not rare to

* *Voyage en Guinée et dans les Isles caraïbes en Amérique*, p. 176. Paris, 1793.
† Prichard: *Histoire de l'Homme, Trad. Française*, vol. ii., p. 3 *et seq.*
‡ *Bulletin de la Société Ethnologique*, p. 77. 1846.
§ A. St. Hilaire: *Voyage dans la Province de Rio Janeiro*, vol. iii., p. 230.

find a fixed form of cranium which, through the absence of foreign marriages, becomes almost special to the tribe. Among civilized nations, on the other hand, and especially in large towns, are found skulls of every shape, even the most opposite from what seems to be the regular type. M. A. Geoffroy St. Hilaire collected in the catacombs of Paris a series of heads of old inhabitants of the town, among which were found all the modifications of which the entire human species is susceptible. The same remark has been made by M. Serres with regard to heads collected in the cemetery of the "Tour St. Jacques." The same observation may be made with the bald; and in our establishment we have found the greatest varieties, from the pyramidal head of the Mongol, to the flattened head of some Negroes. The shape of the skull, which is so varied among Europeans, is not less so among Negroes. Thus Weber, Al. d'Orligny, and M. Parchappe* have come to the conclusion that in no nation does there exist, with respect to the shape of the skull, any fixed characteristic.

One last fact, and with this we conclude our examination of the organic signs supposed to constitute differences between the several races of mankind.

The features have been considered to give a metrical scale of the physiognomy of the different races of man; but careful observation overthrows this obstacle. Blumenbach had already remarked that there are to be found Ethiopians, who, except their colour, have the most handsome features of our species. Prichard has noticed the same regularity in a Negro of Haoussa. This opinion with respect to the beauty of form of certain Negro tribes is also held by Raffanel, Caillé, Claperton, and Barbot. Prichard has in his work a drawing of three heads, one that of a Congo Negro, another that of an American from Louisiana, and the third that of a Chinaman; and there is the closest analogy in shape between the three.

According to Bodwick, the higher class of Ashantees are not only well made, but have features resembling those of the Grecian type: it is a long step from that to the monkey-shaped muzzle usually assigned to the Negro. Livingstone remarks that the Caffre head is as well made as the European. Several of the Bushman tribes, he adds, are on the whole handsome men; and the monuments of the ancient Egyptians show much truer types of the Balondas than any drawings in works on ethnography that have come into my hands.—(Op. cit., p. 194 and 421). This resemblance between the Negro and the Caucasian types tends to confirm the opinion expressed by M. Serres, that each race has in it the germ of the type of other races.

* Parchappe: *Instruction pour le Peuple—Anatomie et Physiologie de l'Homme*, p. 704 *et seq.*

With this fine shape of the head and regularity of feature in a large number of Negro tribes, is it necessary to inquire whether the inferior capacity of the cranium of this race is real? The degree of intelligence shown by these people (of which we shall speak shortly) would be sufficient answer; but anatomical proof has been furnished by Dr. Morton, who measured 286 heads of the different varieties of mankind; and he found skulls of white men with a minimum of seventy-five, and skulls of Negroes with a maximum of ninety-four; whence it follows that some Negroes have greater development of brain than some Europeans.

I will not do more than mention the objections which have been based on the defective junction of the great wing of the sphenoid with the anterior inferior angle of the parietal; from the more backward situation of the occipital foramen; from the structure of the pelvis; from the proportion of the limbs; from the shape of the calf or of the heel; from the darker colour of the blood; from the fetid perspiration; because to all these objections unanswerable replies have been made.

We have shown, then, that none of the characteristics by which it has been attempted to separate the Negro from the Caucasian, are so invariable as they have been said to be. Consequently, we think ourselves justified in coming to the conclusion that the physical differences set down are not sufficient to overthrow the theory of the unity of the human race.

The examination of the psychological characteristics, upon which we shall now enter, will give still greater force to this opinion, which is held by a great number of illustrious men.

But, before entering upon this part of the subject, let us discuss an objection which we find reproduced in the very interesting work of M. G. Pouchet, on the plurality of the human species. There are monuments, says this distinguished observer (probably the tomb of Rhamses Meiamoun), which, dating 3000 years back, prove unanswerably that the most decided transformations were accomplished at that time; and the thousand remaining years cannot explain the transformation of a transplanted race, since, at the end of 500 years, we find it as it was before.* We are not going to enter upon a defence of Bible chronology, but we believe with M. Godron, who has published a book so rich in facts, that the style of living, which changes so powerfully the human species, must have been at work since the origin of different nations; and the changes once acquired being propagated by inheritance, became permanent and uniform through the continuance of the same mode of life and the absence of foreign alliances.

It is, besides, quite undeniable that among domestic animals

* *De la Pluralité des Races—Essai anthropologique*, p. 124. Paris, 1858.

new races may be formed very rapidly, and sometimes even without the interference of man. Less than a century ago there was born in America a bull without horns, and, without any interference in order to propagate this peculiarity, the bull perpetuated his species, and became the stock of a hornless race, (the mocho ox), and spread itself over entire provinces.*

In 1791, in Massachusetts, among the English race of sheep, a ram was produced remarkable for the length of its body, the shortness of its legs, and a trunk like that of a terrier. These circumstances rendered it unfit for leaping the enclosures. In this case man intervened, and, by means of cleverly-managed crosses, these sheep have multiplied, and formed the 'loutre' (*ancon*) race.† When we come to speak of crosses which have lately been made between European and savage tribes, we shall establish facts analogous to these. One more objection might still be made. Why, it may be said, if these changes took place in former days, do they no longer show themselves at the present time? There is no reason, for instance, why the colour of the skin should not undergo a fresh change. We are not in a position to give a satisfactory answer to this question. We will only call attention to the fact, that for some years past partial blue and black discolorations of the skin of the face, especially of the eyelids, have been observed in persons who are in good bodily health. On this, consult a paper by Dr. Leroy de Méricourt. Dr. Hardy, who has just published a new instance of it (*Union Médicale*, Mar. 1860), says that there are already seven or eight cases of it in the town of Brest. The person he describes is in good health, menstruates regularly, and belongs to the middle class.

Without denying the value of M. G. Pouchet's objection, these facts are of a nature to diminish its force in a marked degree. However, we will not insist longer upon this point, but pass on to the examination of psychological peculiarities.

Those who are in favour of a plurality of human races, born in the different spots where they are met with to this day, have not only pointed to the difference in physical characters, but have passed in review the amount of intelligence possessed by the numerous nations of the earth; and have sought to establish that, if several among them are richly endowed, others have for their share but a certain amount of ability; and that some even are completely destitute thereof. According to this, those people, placed below animals—and particularly below the human-shaped ape, which forms a link between man and the animal kingdom—would constitute inferior races, and prove the inequality of the human species. Doubtless, there are stationary tribes, degraded

* Don Felix de Azana: *Voyage dans l'Amérique septentrionale*, vol. i., p. 378.

† *Transactions philosophiques*, p. 58. 1813.

by misery, degenerated by being deprived of the gifts of nature, and by deleterious influences; but does close observation justify the doctrine of the inequality of races, and of the existence of superior and inferior classes of men? Let us examine this question.

There is a savage race in the south of Africa remarkable for the development posteriorly of a protuberance of fat in the females, and, through a kind of clucking which approximates them to the lower animals, out of the pale of those who speak known languages. Certain travellers and anthropologists have placed this race in the lowest scale of humanity, if they have not classed its members among animals. This is the Bosjesman nation, or rather a tribe of that nation, as we shall see presently. These people lead truly a most precarious and miserable life, but they are not so devoid of intelligence as has been affirmed. Péron tells us that De Genssens, Governor at the Cape, had in his house a young Bosjesman who had acquired Dutch and a little English* with the greatest ease. But there is yet room for inquiry into the pretended degraded state of this people. Livingstone, whom we are fond of quoting, because he has seen things without any preconcieved opinions, speaks as follows concerning the Bushmen (Bosjesmans):—"They live in the desert from choice. Many of them are of short stature, yet without the deformity of dwarfs. Those who are brought to Europe have been chosen on account of their extreme ugliness. In the suburbs of Zambo, the Bushmen are in general handsome men, well-made, and with an independence of spirit almost absolute"—(p. 194). A conscientious observer, who has studied with the greatest care the comparative anatomy of the brain, has determined that this organ in the Bosjesman, without being so voluminous and complete as that of the European, is in every respect similar to a human brain. He therefore looks upon this people as susceptible of intellectual development. Among small tribes like the Bosjesmans, the convolutions of the brain are but slightly developed; for instance, the brain of the Hottentot Venus, of which M. Gratiolet has a wax model, presents a degree of simplicity which in white nations corresponds to idiocy. Yet this woman was anything but an idiot.†

The Australian, placed in the same catalogue, ugly, thin, and ill-formed, has also been looked upon as a brute. But the cruel extremities to which they had been reduced were forgotten. Driven by the English from the beautiful districts which the latter have covered with thriving colonies, the natives of New Holland were obliged to take refuge in the interior of Australia. A dry country, vast deserts of sand, thickets where no water and

* *Voyage aux Terres Australes*, vol. ii., p. 311.

† *Moniteur des Sciences Médicales et Pharmaceutiques*, p. 103. Feb. 1, 1860.

scarcely any game is to be found, and in consequence frightful famine and privations of every kind—is there not in such a combination of circumstances sufficient cause for degeneracy? Yet Prichard tells us that Australian children, who have been adopted at Port Jackson, have learnt to read, write, and draw as well as white children of the same age.*

The apparent inferiority of these two tribes, the tales told second-hand of several naturalists, who declared they had seen, on the northern coast of New Guinea, trees swarming with natives of both sexes leaping from branch to branch like monkeys, with their weapons slung on their shoulders, gesticulating, shouting, and laughing;† and similar observations said to have been made in the forests of India, ought to strengthen the opinion, they say, that the ape belongs to the order of bipeds. Richard Owen, also, one of the most celebrated anthropologists of our time, has not hesitated to say that the distinction between this animal and man is the stumbling-block of anatomists.‡

The ourang-outang has naturally been held up in opposition to these so-called inferior races by the partisans of the animal kingdom. Its quickness in imitating man, its adroitness in many things, its affections, passions, intelligence, which, not to underrate it, seemed to want nothing but language, made it an intermediate being between the two. "It is neither a man nor a monkey," said the crowd that gazed at the chimpanzee in the Jardin des Plantes; and this is the theory held by the celebrated Geoffroy Saint Hilaire. We cannot agree with this opinion, as M. Gratiolet has clearly demonstrated, that the brain of the ape has anatomical characters which entirely separate it from that of man. In the monkey, the middle lobe begins and ends before the frontal lobe; with man, on the other hand, the frontal convolutions appear first, and those of the middle appear subsequently. Consequently the brain of man differs from that of the ape the more in those of recent formation, and an arrest of development can but exaggerate this difference.

But without insisting upon the difference in gait, on the length of the upper limb, the make of the hand, or the absence of speech, there is one characteristic which separates the ape as well as the rest of animals from the human race; and that is, their non-progressive state. During the thousands of years that animals have been in contact with man, they do no more than they did in the first instance, or than what they have been taught to do. The nests built by the beaver or the bee are the same as were described centuries ago. Their sociability and their fitness for education

* *Hist. Nat., op. cit.,* p. 266.

† Crawford: *British Association for the Advancement of Science,* p. 8. 1852.

‡ *On the Characters of the Class of Mammalia,* p. 20. 1857.

are just what they were at the commencement; while man, placed in the most disadvantageous condition, is susceptible of education and of perfection. This we have just shown, and shall illustrate by instances still more conclusive.

There is one race of mankind which has been specially the object of the most violent attacks, and which polygenists have declared to be incapable of amelioration. You will not be surprised that an American should have written these words: "Show me a single line written by a Negro worth remembering."* Facts will show how erroneous this opinion is. Look over the documents laid before the English Parliament on the 19th of May, 1829, and you will there find repeated proofs of the immense superiority in intelligence of children born of emancipated Negroes in the colony of Sierra Leone over those born of negroes still in a state of slavery, though living in the same colony. Two years ago, a Mulatto and a Negro carried off high prizes at the general meeting at Paris, and this is not an isolated case. The journal *Le Propagateur de la Foi* announced that twenty black missionaries were preparing to carry religious instruction into savage countries. The *Revue des deux Mondes* gave us, some years back, details full of interest respecting the literature of St. Domingo. The Academy of Science counts among its correspondents M. Lillet Geoffroy, a negro well versed in mathematical science. Livingstone tells us that Negroes learn the alphabet in a few days. He was struck with the knowledge of the Ambakystas, nearly all of whom can read and write with remarkable facility. They learn with eagerness everything they can—history, jurisprudence, &c. —and from their tact for commerce they have got the name of Anglo-Jews. Among the Makololos no individual has the slightest influence if he is not of irreproachable morals, and has not a loyal heart. Every kind of immorality is severely condemned by these idolaters. (p. 543.) My opinion of the Negro race is the more impartial, seeing that my grandfather died at St. Domingo, and his goods were confiscated. The conquest of Africa begins to bear fruit; the Arab tribes, who appeared to be so hostile to European civilization, begin to appreciate the advantages of a home, and houses begin to spring up. In Algiers, Arab children go to school, and are remarkable for their quickness. The commandant De Martimprey lately reviewed the young native sea-boys destined to furnish France with sailors. He was struck with their progress, and expressed his pleasure thereat. This institution, which is perfectly well received by the Arabs, receives a great number of applications for admission from the Arab families.

The same triumphs of civilization are found in America. The

* Gliddon: *Types of Mankind*, p. 56.

Indians had been proclaimed unteachable outlaws, only fit to be exterminated. How did they answer this cruel description ? One of their tribes, the Cherokees, settled some years ago in the north of the Georgia, Alabama, and Tennessee States, built houses, worked, and tilled ; and these natives, who, at the commencement of their new life, were reduced to 5000, are now reckoned at 15,000 inhabitants, in good circumstances.

The *Moniteur Universel* of the 7th October, 1858, published the following notice of the Veddahs of the island of Ceylon : "This tribe, whose forefathers were the first Buddhists,* had sunk to a state of thorough debasement. They dwelt in the mountains, living on wild honey and the produce of fishing. Mr. Mackenzie, the governor of the island, in pity made the first overtures in their favour. Two villages were built, and the Veddahs were invited to come and live in them. Some of them did leave the horrible caves and pitiful huts which they had made their dwelling-places. They were persuaded to take to agriculture. Aided by the English Government, the little colony soon grew, and prospered day by day. At the present time the greater part of the Veddahs profess Christianity."

Lastly, there was a short time back a curious article in the *Quarterly Review*, upon the amelioration of the inhabitants of New Zealand. Scarcely a century ago, this colony, situated in the middle of the Pacific Ocean, was still looked on as a wretched country, peopled only with savages, who were in the habit of plunging into the debauchery of cannibal feasts, the last of which took place in 1842. By placing these natives under the protection of the law, England raised the level of these races. Civilization, the precepts of Christianity, and material progress exercised upon them the happiest influence. Life and property are at the present day as secure in New Zealand as in the mother country.

Many European villages have attracted into their neighbourhood, or include among their population, a considerable number of Maoris, who are united by the same interests, hold the same faith, and stand in the mutual relation of landlord and tenant. The writer of the article adds that the progress made by the natives in agricultural science and rural economy is truly surprising : and the chances of the harvest form now the chief interest of savages who were once so warlike and so cruel.

If the development of material interest carried to excess has been the subject of well-earned reproach, we must also acknowledge that it has helped to propagate and introduce ideas of amelioration which were unknown to people who were beyond the circle of

* Philarète Chasles : Morale Chrétienne des Boudhistes. *Débats*, April 24, 1860.

intellectual progress. Little space would suffice to show the influence which the prolonged stay of our soldiers has had upon other populations.

The few facts which we have gathered together then show that man is susceptible of amelioration, even when he is found in a marked degree of inferiority, in comparison with our own race. However low, therefore, a nation may have fallen, we protest against the opinion of a certain class of economists, who make a sweeping condemnation of certain races which are destined, according to their idea, to disappear from the face of the earth on account of their irremediable inferiority. No, indeed: as Christians and philosophers believing in the unity of the races of mankind we cannot sufficiently condemn such a doctrine. All that is necessary in such a case is to have recourse to all suitable means to raise them from their decay. "Homo sum, humani nihil a me alienum puto," wrote an ancient author; such is our motto for all. These thoughts, which occurred to me on seeing the work by M. d'Escayrac de Lauture upon Turkey, are in accordance with the motto of his book, "Aperire viam gentibus:" and the curious circumstances of resemblance between the Turks and the Franks which he quotes prove that intercourse only is necessary to multiply them.

If man is distinguished from animals by his psychological characters, which are more or less marked in different races, but in all instances susceptible of development and perfection by ameliorating their physical condition, the moral characters, intercourse, example, and education, play no less a part in drawing the line between the two species. Of these characters, there is one in particular which by its generality, I may say its universality, is the exclusive property of the human race. I mean devotion to one's fellow-beings. Wherever there is a sufferer, be it Australian, Negro, or even animal, there is a score of generous hearts ready to give succour. This feeling is so deeply rooted in man, that in time of pressing danger it will come to the help even of an enemy. In order to be useful to others and help them to share in the general welfare, man will sacrifice his rest, his property, his fortune, even his life. This devotedness on behalf of the masses so long in wretchedness, which spreads more and more among the enlightened classes, is a divine mark of our species, which cannot be explained by causes arising from our organization, and whose origin must consequently be sought for in a principle of another nature. To suppose that the Alpha and Omega of things are inaccessible to us, is to run counter to the very notion of cause, which is foreign to the philosophy of sensation.

We have abstained, in this examination of organic and psychological characteristics, from making any allusion to dogmas which we respect. But ought we to follow the advice given by a man of feeling and talent, to banish thoughts of equality and fraternity because their interference might be prejudicial to science? That is an opinion which we cannot share, and we think we shall not err in saying that the Society will not share it either. However interesting science may be, we think it is only useful so far as it serves for the benefit of mankind; and as soon as it ceases to have this end, it no longer deserves the labour of students. In this respect, we agree entirely in the opinion expressed by the writer of the article on the *New Theory of Natural History:* "Wherever slavery oppresses a perfectible moral nature, or a free will capable of being guided by conscience and religion, it is a crime and a monstrosity; this is a truth to which every honest mind ought to cling, and which is more durable than all the other doctrines of ethnography and natural history which may be in the ascendant to-day and overthrown to-morrow."*

While upholding the doctrine of the unity of the human race, we reject, with Al. von Humboldt, as a matter of course the unhappy and unproved distinction made between superior and inferior races. Doubtless there are some more susceptible of culture, more civilized, more enlightened, but none more noble than others. All are made equally for liberty, and the instance of the New Zealand savages, once cannibals, now agriculturists and landlords, is the best of all proofs.

Let us here stay to examine an important objection arising from the study of philology. It cannot be denied that the grammar of a language is its code of rights, and when we cannot logically derive one language from another, nor refer two dialects to a common stock, it seems natural to conclude that the two languages are not of the same family. It is clear that the Indo-European languages spring from one common dialect, now dead. They all have, for instance, the same form of the verb "to be;" the form which is also found in the Sanscrit. On this subject, it will be found interesting to read the lecture given by M. Monlau as his introductory discourse befo e the Spanish Academy. But in our actual state of knowledge on the subject, can we show parallel resemblances between the Sanscrit, the Semitic languages (Hebrew, Chaldaic, Arabic), Chinese, and the American idioms? At the commencement of our medical studies, we followed the learned instructions of Professors Abel Rémusat, De Chézy, and Caussin de Perceval: and we confess that the grammatical differences of these languages appeared to us so decided, that in order to find for them a common origin it was necessary to suppose the most extraordinary transformations. It is possible, as

* *Revue des deux Mondes,* p. 467, April 1, 1860.

M. Revau says, in his remarkable *History of the Semitic languages*, that the Assyrian preserves an intermediate dialect which would form a link between the Sanscrit and the Hebrew. Yet it may well be declared that the unity of human languages is not yet satisfactorily demonstrated.

This objection is a grave one, and we recognise it at once: but is it really worth the stress that has been laid upon it? For, since it is clear that the dialects of an advanced civilization have been lost in Eastern countries, we do not possess all the elements necessary to the solution of the question: and it only wants a new Anquetil-Duperron, or a second Burnouf, to find the key to the cuneiform hieroglyphics, and so overthrow the whole system. And we know well that in science what does not exist to-day may exist to-morrow.

In the examination which we have just made of the characters of organic life and of intellectual life, we have striven to assign to each of these elements the position which it should occupy. If we admit, in accordance with repeated observation, that the first drop of blood in man contains in an undeveloped state his physical and moral qualities, as well as his ills and vices, we are no less strongly convinced that there is something superior to the drop of blood. Doubtless nations, like individuals, possess different abilities and special powers; and history compels us to admit that, whatever be the reason, the races of men have not all the same amount of intelligence, the same moral vigour, the same forcible inspiration towards the ideal. The mission of some appears to be war, of others sociability; and of several, art—

"Excudent alii spirantia mollius æra...

Tu regere imperio populos, Romane, memento;

Hæ tibi erunt artes."

While admitting these differences, says M. Gustave de Beaumont, of a secondary nature, we must never lose sight of those grand traits of generosity which are common to all men and to all nations. Just as all human beings experience the same material appetites, which constitute one of the conditions of physical life, so all are endowed with certain immaterial faculties which form part of their moral existence. All possess the instinctive love of liberty and of acquiring property; of liberty, which is the use of one's body, and of the acquisition of property, which is the expression of one's wants. Some are born by the chance of circumstances in a state of freedom, others in slavery: some with blessings of which others are deprived. The first lose by their vices what the second have the merit of creating: but all are glad to possess, and all suffer by being deprived of these blessings: all equally enjoy, desire, or regret them. Let egotism deceive itself with regard to these truths, and obscure them; but let not science intervene, and be called to the aid of errors which she combats and of lies which she disavows.*

These immaterial faculties which are found in all men are a new argument to be added to those furnished by the study of psychological characters, and they establish, in our opinion, incontestable proof of the unity and speciality of the human race.

We have now arrived at the last portion of our task, which has not been less frequently called in question than the foregoing, but which appears to us as true, and perhaps even better proved, than the other questions; I mean the formation of races and their crosses. The formation of races is a consequence of our nature. As soon as the family increased, the diversity of inclinations, instincts, and passions, the thirst after independence and the necessities of life, brought about a separation. Subject to influences, among which the mode of life and internal causes occupy the chief place, man changed the more quickly in proportion as he was nearer to the original stock; and we must not forget those external influences which act all the more strongly in proportion as man is less civilized. Some have denied the rapidity of these changes; but those which have occurred in our own times justify this view of the case. The Society will remember the rapid appearance of the "Mocho" and "Loutre" races. The same facts are observable in the human race.

The American race, which owes its existence to the English nation, from which it has been separated scarcely a century, presents nevertheless such striking differences in physical, physiological, and psychological relations from the latter, that Dr. Knox thought himself justified in concluding that they are a kind of degenerated type of the mother country. Among the psychological characteristics, there is one which has specially struck me. While the Englishman, shut in his "home," scarcely opens his door to look at a foreigner (which gave rise to Chateaubriand's remark, that an exile may be next door to an Englishman for whole years, and learn nothing of his habits, manners, or mode of life), the American receives all foreigners with open arms, and assimilates them so rapidly to himself, that we have known Englishmen who started with all the prejudices of their country, return in two or three years more Americanized than the Americans themselves.

The dispersion of the human family into an infinitude of fragments, tribes, and societies, produced mixtures and crosses more or less numerous, of which it is important to inquire the result.

Before pointing out the principal results of crosses between different men, it is necessary to explain in a few words the plan

* Gustave de Beaumont: La Sóciété Russe et la Sóciété Américaine. *Revue des deux Mondes*, p. 1182, March 15, 1854.

adopted by intelligent breeders to modify and reform races of animals. Their first object is to discover with tact characteristics which are susceptible of regular transmission; for, as M. Auguste Laugel observes, it is by regulating attentively the succession of generations, that step by step the required object is attained. And the definite result includes the sum total of all these steps. This procedure is called "selection." In Saxony, the importance of this principle is so well understood with respect to the merino sheep, that selection has there become a trade. The sheep are set upon a table, and studied as a connoisseur studies a picture. This is repeated every month, and each time the sheep are marked and classified; and the best only are definitely chosen for breeding.* It is partly to this kind of proceeding, says M. Edward Milne, in his *Traité de Zoologie*, that the Arab horses owe their well-earned reputation. The Arabs attach such importance to the purity of their splendid horses, that their pedigree is always authenticated by official documents. They count the family of some of these noble animals backwards two thousand years; and there are a few whose lineage may be shown to extend over a period of four centuries.

We have given these particulars thus detailed, because we wish to establish the superiority of the means commonly employed to preserve cross-breeds among animals; while those among man are the result of chance, and have not yet among common people been made a subject of reflection, or of any particular method.

It is well known that when a cross is made between two animals of the same species, the offspring takes after both parents, but generally inclines to the father; hence a male of the purest breed is employed to ameliorate an indifferent breed. We also know that by breeding with this offspring, and avoiding mixture from other species, a mongrel race is procured, which at last acquires a certain stability and uniformity; but that if the product of the first cross is put to breed with one or other of the original parents, the offspring returns to the original type.

The crosses between races of man, whether between neighbouring or distant people, follow the same law of variety; on the one hand, returning to one of the parent stock, or on the other, producing mixed races according to the nature of the successive crosses. The first result is clearly seen in the offspring of the European with the negress, *e.g.*,

| | | |
|---|---|---|
| White and black produce the mulatto. | | |
| White and mulatto | „ | quadroon. |
| White and quadroon | „ | quinteroon. |
| White and quinteroon | „ | white. |

* Auguste Laugel: Nouvelle Théorie d'Histoire Naturelle, l'Origine des Espèces. *Revue des deux Mondes,* April 1, 1860.

And *vice versâ*,

| | | |
|---|---|---|
| Black and white | produce the | mulatto. |
| Black and mulatto | „ | griffon and zambo. |
| Black and griffon | „ | zambo prieto. |
| Black and zambo prieto | „ | black. |

It is thus shown that after four generations the mulatto becomes lost in one of his original stock.

These circumstances have been brought about on a large scale. Thus, the first Chinese who came to inhabit Malacca, having brought no women of their own, married Malays. To the present day these families make no alliances except among themselves, or with Chinese who come over from the mother country. The results of the strict observance of this custom has been to produce women exactly resembling those at Fokin or Canton.*

The production of new species among domestic animals is an undeniable fact; and the opponents of the doctrine of unity have sought to explain it by the demoralizing tendency of a state of servitude. This argument must surprise us when we see the magnificent exhibitions of horses and animals of all kinds which are the delight of connoisseurs. Besides, we might answer by applying the argument to the human species. The adversaries of our doctrine do not confine themselves to this criticism; they deny the existence of a mongrel race of men, and hold that such a race can only be perpetuated by the continued presence of two original types; and they say that, owing to the natural tendency to return to the primitive stock, and the usual barrenness of these half-breeds, the mixed race must be always inferior in quality and number. We will not go far to find a peremptory answer: M. Broca supplies one in the case of the French nation. But before giving it, we must not lose sight of the fact so judiciously pointed out by M. W. Edwards,† that conquering colonists, unless they imitate the Jews or the English in India, become in the end lost in the conquered race. This happened with the Romans and Francs, whose type has almost entirely disappeared in the Gauls: while the types of the Gauls, Gaëls, or Celts, or of the Kimri or Cimbri, who were the former rulers of this country, have been perpetuated, and produced a cross race, which has suffered no loss of fruitfulness, energy, or intelligence; this was shown by Dr. Broca at one of the meetings of the Anthropological Society. The Gauls, better known under the name of Celts, were a race of small men, dark-complexioned, with round head, broad forehead, not prominent nose, rounded face, and hairy body. The Kimri or Cimbri were tall and fair, with a

* Docteur Yvan: *De la France en Chine*, p. 237. Paris, 1855.

† *Des Caractères Physiques des Races Humaines, considérées dans leurs Rapports avec l'Histoire*, p. 40. Paris, 1829.

long head, high forehead, long, prominent, hooked nose, protruding chin, and short hair. The Kimri occupied the north-east; the Celts the south, centre, and north-west. The Kimri element predominates notably between the Seine and the Rhine; while south of the Loire and in Brittany the Celtic element preponderates.

In the difficulty caused by the numerous mixtures which have resulted from the crossing of these two races, M. Broca had recourse to the distinguishing mark afforded by the difference in height and make, which is valuable, on account of the positive evidence afforded by the conscription. The result of his inquiries is, that the average height is greater in the Cimbric than in the Celtic parts. And he shows that the effect of the cross has been to raise the average height of the Celts and to lessen that of the Kimri, and that the departments where the average height is lowest are those where the Celt has undergone the least amount of crossing.

Another conclusion to be drawn from this study is that these crosses have not had any deleterious influence on the population; for the strength, health, fruitfulness, and longevity are the same in the average, whether the races have been little or much mixed. Here then is an experimental proof—that a cross between two races of the same group produces a perfectly healthy population, which propagates itself without returning to the stock of either of the original races, and in no respect second to them in physical and intellectual qualities. Must we then admit, on the other hand, that crosses between very distant races are unproductive or can only produce half-breeds of diminished fruitfulness? But experience on this point has also been had on a large scale in the European colonies, and the half-breeds who owe their existence to it are now very numerous. In the five States of Mexico, Guatimala, Colombia, Plata, and Brazil, they form a fifth of the population.* Omalius d'Halloy estimates the whole number of the population of the globe at 750 millions, and that of the half-breeds which have been formed since the great movement of the 15th century at 10 millions.

After the conquest of America, the Spaniards mixed with the natives, and their children, or bastards, were called Spaniards. These, says Felix de Azara, united, and their descendants form at the present day in Paraguay the greater number of those who are called Spaniards. They seem to have some superiority over the Spaniards of Europe in regard to height, elegance of form, and fairness of skin. These examples of the crosses between different races are not the only ones which solve the important problem of continued fruitfulness.

* Quatrefages: Histoire Naturelle de l'Homme. *Revue des deux Mondes,* 2me période, vol. viii., p. 162.

Wherever correct observations have been made, the half-breeds have shown themselves superior in some respects to the white race itself. In the Philippine Islands the half-breeds are very numerous, and form an active, industrious, and brave class, which has already wrested important and just concessions from the metropolis. It is scarcely necessary to recal what kind of men those were who were so cruelly cut up by civil discord in St. Domingo. In Brazil, the cross-breed between black and white has been enabled, thanks to its intellectual and moral worth, to conquer in a great degree the prejudice of blood; and it is a race peculiarly remarkable for its cultivation of the arts, which are much more developed among them than among the pure whites. In the same empire we find an entire province crossed between Europeans and natives. What is the result? The peculiar features of the Paulistas, their chivalrous character, their bravery and perseverance, have been noticed in good works written by reliable authors.* A short time back, the *Quarterly Review* quoted a very interesting example of this crossing. The islanders of New Zealand had lived for centuries like true savages. England has made them citizens, and they adopt the customs of the mother country. Marriages have taken place between Europeans and New Zealanders. The produce of this is about 500 individuals whose natural superiority is undeniable.†

M. Gratiolet remarked at the meeting of the 14th of March, that in order to study primitive stocks and races of men, we must as soon as possible study savage tribes which are as yet free from alliance, because cross-breeds were becoming so common that primitive races would soon become extinct. The following remarks which we extract from the *Revue des deux Mondes*, show the rapidity with which practical ideas make their way:—

"The utility of crossing, in order to ameliorate a race, has not escaped the notice of savage nations. The Goajires of New Granada, according to M. Elisée Reclus, are extremely handsome, and are formed with sculptural beauty; their faces are generally round, their colour, which is red in youth, gets darker as they grow older, and, in old age, they are a fine mahogany colour. Among these men, the true aristocracy is that of beauty; riches and power belong to those whom nature has favoured in this respect. If a shipwreck throws foreign sailors on their coast, these Indians, who know the 'callipedic' importance of a well-arranged cross, retain those who are tall and vigorous, and make them pay for the hospitality granted them by a few years of forced marriage with two or three handsome 'Goajires.' Those unfortunate sailors who happen to be ill-made, are stripped of their clothes, and turned over from tribe to tribe as far as Rio Hacha, hooted and ridiculed."‡

* M. Quatrefages, *op. cit.* † *Monit. Universel*, 11, 14, 23 Jan. 1859.

‡ *Revue des deux Mondes*, vol. xxvi. pp. 437, 438, March 15, 1860.

In spite of the vices and defects which they have in common with other barbarous nations, the aboriginal Indians are making evident progress; and there is reason to believe that in the province of Rio Hacha they will form, as the Indians of the interior have formed at Socorro, Velez, and Pamplona, the most important element of social regeneration. Up to the latest times they kept themselves free from all intermixture; but the numerous opportunities for intercourse resulting from commercial relations have lately produced some remarkable families of cross-breeds. Already the commerce between Goajire tribes and foreigners is larger in proportion than that of any other community of the Granada republic. Many Goajires have lately settled here and there on the right bank of the Rio de Hacha, and have cleared the land preparatory to planting mangoes and other fruit-trees. Five or six families, attracted by the hope of gain, have gone a step further. A short distance from the town they have established market gardens in sufficient number to supply the town.

One last trait in the character of the Goajires is the hatred which they, in number about 25,000 or 30,000, cherish against the Spaniards, and the vengeance which they have exacted in course of time. For nearly three centuries these aborigines have waged war against their conquerors, who, besides conquering them, used to behead them, cut them in pieces, feed dogs on their flesh, and reduce them to slavery. The continued war which they have carried on against the descendants of these Spaniards has been so terrible, that the latter have completely disappeared from this part of New Granada, and no Spaniard dare trust himself on the other side of the Rio de Hacha. This is a lesson which ought not to be lost.

M. Elisée Reclus relates another fact respecting the relation of races in New Granada on the Sierra Negra coast, one of the great chains of the Andes. The vast plain of Rio-Caser has as yet on it only a few scattered villages; before long it will resemble our own country. The agents in this change will consist to a great extent of emigrants from Europe and North America. But the Indians of the Sierra, the Tupes, the Arŭaques, and the Chimilas, will also play an important part therein. A few years ago the Chimilas were still deadly enemies of the Spaniards and of coloured men. Covering their bodies with bark stripped off the trees, they lived in the grottoes and forests round Cerro-Pontado, and any foreigner who ventured near their retreat was murdered without pity. One day a negro of Herculean strength, named Christoforo Sandoval, instigated by some strangely bold fancy, went and presented himself before the chief of the Chimilas, unarmed, and accompanied only by his young son. By what magic the negro succeeded in charming the Red Skin we know not, but the effect was instantaneous; the Cacique abdicated, and Cristoforo took his place as chief of the Chimilas. From that day the Indians made peace with the Spaniards, and turned their attention to agriculture.

M. de Rochas, a navy surgeon, who has published an excellent pamphlet on the anthropology of New Caledonia, after observing that the New Caledonians, who belong to the Oceanic Negroes, have a dirty black skin, something of the colour of chocolate, points out an improvement in form of some of the tribes of the eastern coast. He is inclined to attribute it to an intermixture of races resulting from emigration from Polynesia. It is certain that, not long since, emigrants from Ouvéa settled on one of the Loyalty Islands (in New Caledonia), conquered the inhabitants, and imposed on them their own language and the name of their own native place. This is the "Halgan" island of Dumont d'Urville's maps, called by the natives Ouvéa. The race of new inhabitants mixed with the old race, and the result was, a population of much finer men than those of the neighbouring islands, at the expense of the yellow Polynesians who had emigrated from Wallis, (or Ouvéa), and to the advantage of the black natives. These half-breeds, whom we may call new, since they have existed for only five generations, are taller and stronger than the Caledonians; their face is masculine and agreeable; their hair flat, or curled in long ringlets, but never frizzly; their lips comparatively thin and but slightly turned up; prognathism little marked; forehead high and slightly protruding; the nose longer and the cheek-bones much less prominent than their neighbours, and their skin much less deeply marked. These details are very useful, because they show that the Caucasian race is not the only one that has the power of regenerating a species. One observation of M. de Rochas, which has also some bearing upon the question of the amelioration and civilization of races, is that which treats of diet. The necessity for animal food is so decided among the Caledonians, that one hears them say, "We want flesh—we must fight." This terrible but forcible language is the result of the weakness which is the result of scarcity of meat. M. de Rochas appears to us, therefore, to be in the right when he says that "the shepherd who shall teach them to rear flocks of sheep will do more for civilization than all the moralists in the world;" and that "the man who facilitates their means of profiting by keeping sheep will have deserved well of France and of humanity."*

Lastly, if an anecdote told by a serious newspaper may be allowed in so grave a debate, I can tell you that a merchant at

* V. de Rochas, Chirurgien de la Marine : *Revue Algérienne et Coloniale, Gazette Médicale, Anthropologie de la Nouvelle Calédoine*, March 31, 1860.

Graham's Town, Cape of Good Hope, sold in two weeks a hundred-weight of steel bands among the coloured ladies of the district (descendants of Hottentots and Europeans), among whom crinoline is in high request.

This enumeration of different facts connected with the subject of crossing between different tribes of men, whether of neighbouring or distant classes, has taught us that the progeny is generally superior to the original types; and teaches us also that degeneracy may be combated by this powerful means. The extent of this paper will not allow us to dwell on this important subject, and we will content ourselves with observing that the examples among domestic animals are conclusive. Without going out of France, and keeping to recent experience, we will instance the Charmois sheep and the Boulogne pigs. By means of a well-arranged cross between the races of Berri and Touraine, and then between the offspring of this cross and the merino ram of New Kent, and uniting these with the inferior ewes of Limousin, a breed has been obtained of twice the value of that so sought after in England. With respect to the Boulogne and Montreuil pigs, they come of a local breed which had much degenerated, but was restored by a cross with the Yorkshires and New Leicesters. The stock thus obtained was bred from, and the result was a superior race, which keeps up annually a brisk trade. With regard to the objections that have been made to crossing a breed, it will be sufficient to say that want of success has resulted from inattention to the most elementary laws of physiology; and this happened particularly when an attempt was made to mix the blood of the English horse with all our breeds of horses.

However cautious we may be in drawing comparisons between men and animals, we think the subject is worth consideration; since, on the whole, the physical organization and physiological functions of the two species are very analogous.

The task I have undertaken is finished. In studying the question of the Unity of the Human Race from an anthropological point of view, I have endeavoured to defend by scientific arguments a noble cause, which had been long ago defended religiously and devotedly at every sacrifice.

If I have succeeded, in presence of the sad strife against liberty and in behalf of slavery, in proving, by facts drawn from physical organization and physiology, the legitimacy of the doctrine of the human family, the proof of which exists in the family itself, —if I have placed beyond doubt the principle of equality and reciprocity of the races of mankind, I shall be sufficiently recompensed for my labours, and for the numerous researches which my want of knowledge on these matters has compelled me to make.

M. Lamarche, advocating also the doctrine of unity, has treated the subject with the authority of his talent and of the experience derived from his long course of political, philosophical, and moral studies.* We cannot follow him upon that ground, which is forbidden to us. Our field is less extensive; but we are both children of that France which has never shrunk from shedding its purest blood in defence of the rights of humanity. Under the flag which she now bears so high aloft, captains and soldiers must close their ranks, and yield each other mutual support, and fight according to their ability. This is what is expressed by Dr. Livingstone, in the following terms, at the end of his work which has been so ably translated by Mdme. Loreau:—"Each one in his place, wittingly or unwittingly, accomplishes the wish of our Father who is in Heaven; the man of science, when he discovers hidden laws whose application draws people to one another, and cements their union; the soldier, when he fights for right against tyranny; the sailor, when he rescues numerous victims from the insatiable greed of soulless traffickers; the merchant, by circulating his country's produce, and teaching nations that they depend on one another:—all working for the amelioration and well-being of their fellow-creatures."

---

### Art. III.—THE STATUS OF CRIME IN 1859.

* On the day on which the proof-sheets of this work were sent me, I heard of the death of this excellent man, who had been the promoter of these inquiries. It was, in fact, from hearing him express his opinions respecting the Unity of the Human Race, at the Medical Society at the Pantheon, that I gathered my materials. After my lecture, he expressed to me warmly his intention to make use of my documents in the new edition of his work. I take this opportunity of acknowledging his kindness, and I regret him the more that there is among the press but one opinion concerning the virtues of M. Lamarche.

